# Supplementary material for: MCL1 may not mediate chemoresistance
Source: bioRxiv. 2025 Aug 27:2025.08.22.671824. Preprint. [Version 1] doi: 10.1101/2025.08.22.671824 (PMC12407772; doi:10.1101/2025.08.22.671824)
Supplement: Supplement 1 [file media-1.pdf]

SUPPLEMENTAL INFORMATION

MCL1 may not mediate chemoresistance

Kylin A. Emhoff,[1] Kunho Chung,[1] Dongmei Zhang,[2] Belinda Willard,[2] Timothy Chan,[1,3] Babal Kant Jha,[1,3] Shaun R. Stauffer,[3,4] Jesse A. Coker,[3,4] Jan Joseph Melenhorst[1,3]

[1]Center for Immunotherapy and Precision Immuno-Oncology, Cleveland Clinic Research, Cleveland, OH
[2]Cleveland Clinic Proteomics and Metabolomics Core (RRID: SCR_026563), Cleveland Clinic Research, Cleveland, OH
[3]Cleveland Clinic Lerner College of Medicine of Case Western Reserve University School of Medicine, Cleveland, OH
[4]Cleveland Clinic Center for Therapeutics Discovery, Cleveland Clinic Research, Cleveland, OH

Correspondance: cokerj2@ccf.org; melenhj@ccf.org

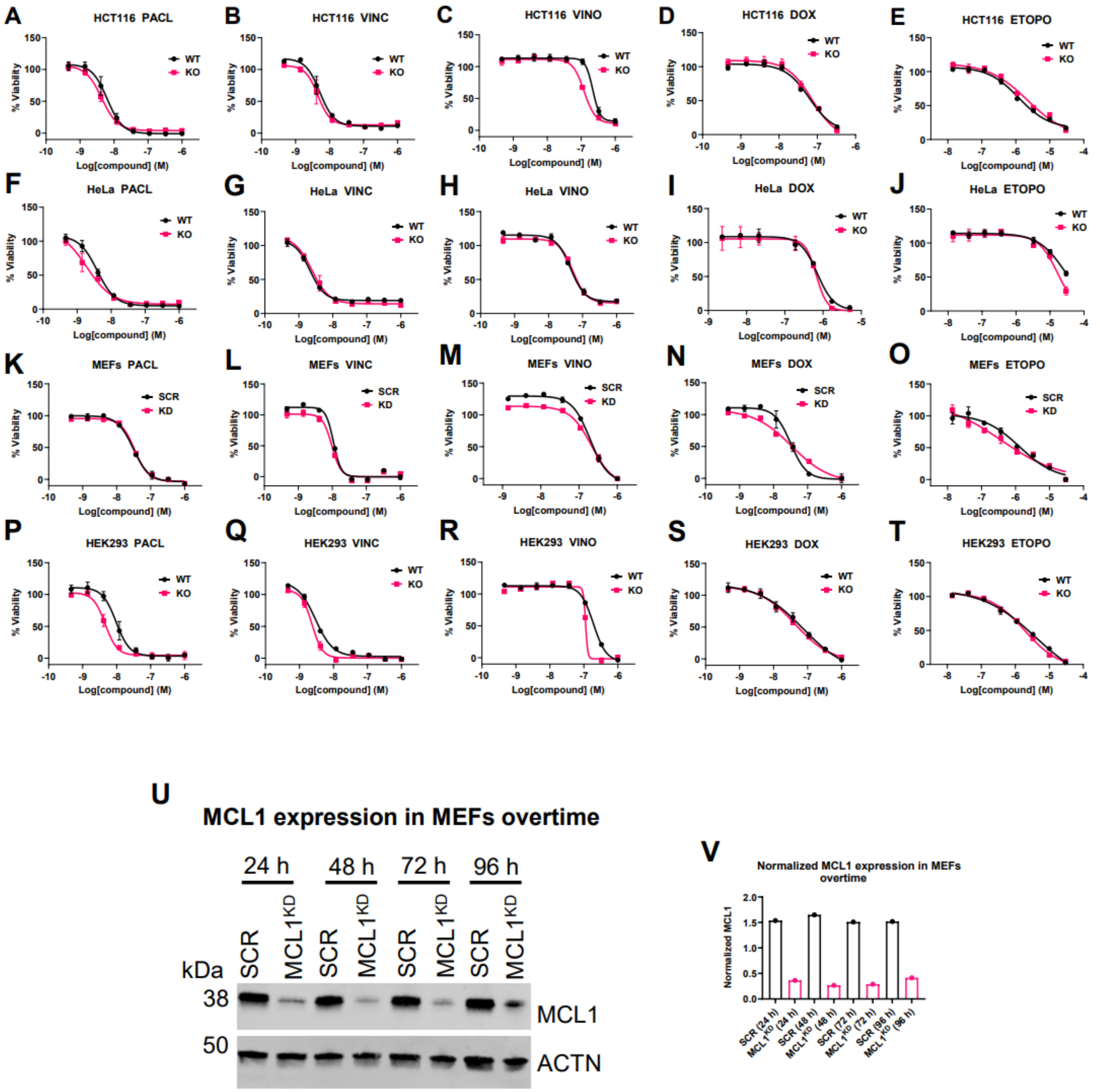

Figure S1. Chemosensitization studies on WT vs. MCL1$^{KO/KD}$ cell lines by CTG®. Cell viability was quantified with CTG® after 48 h treatment with paclitaxel (PACL), vincristine (VINC), vinorelbine (VINO), doxorubicin (DOX), or etoposide (ETOPO) in **A-E)**. HCT116, **F-J)**. HeLa, **K-O)**. MEF, and **P-T)**. HEK293 cells. Assays were performed in biological triplicate, with each one in technical duplicate. All data points on viability curves represent average % viability ± SD. **U-V).** MCL1 expression monitored over 4 days in MEFs after MCL1 siRNA transfection; data are representative of two independent experiments. SCR = scramble siRNA (negative control).

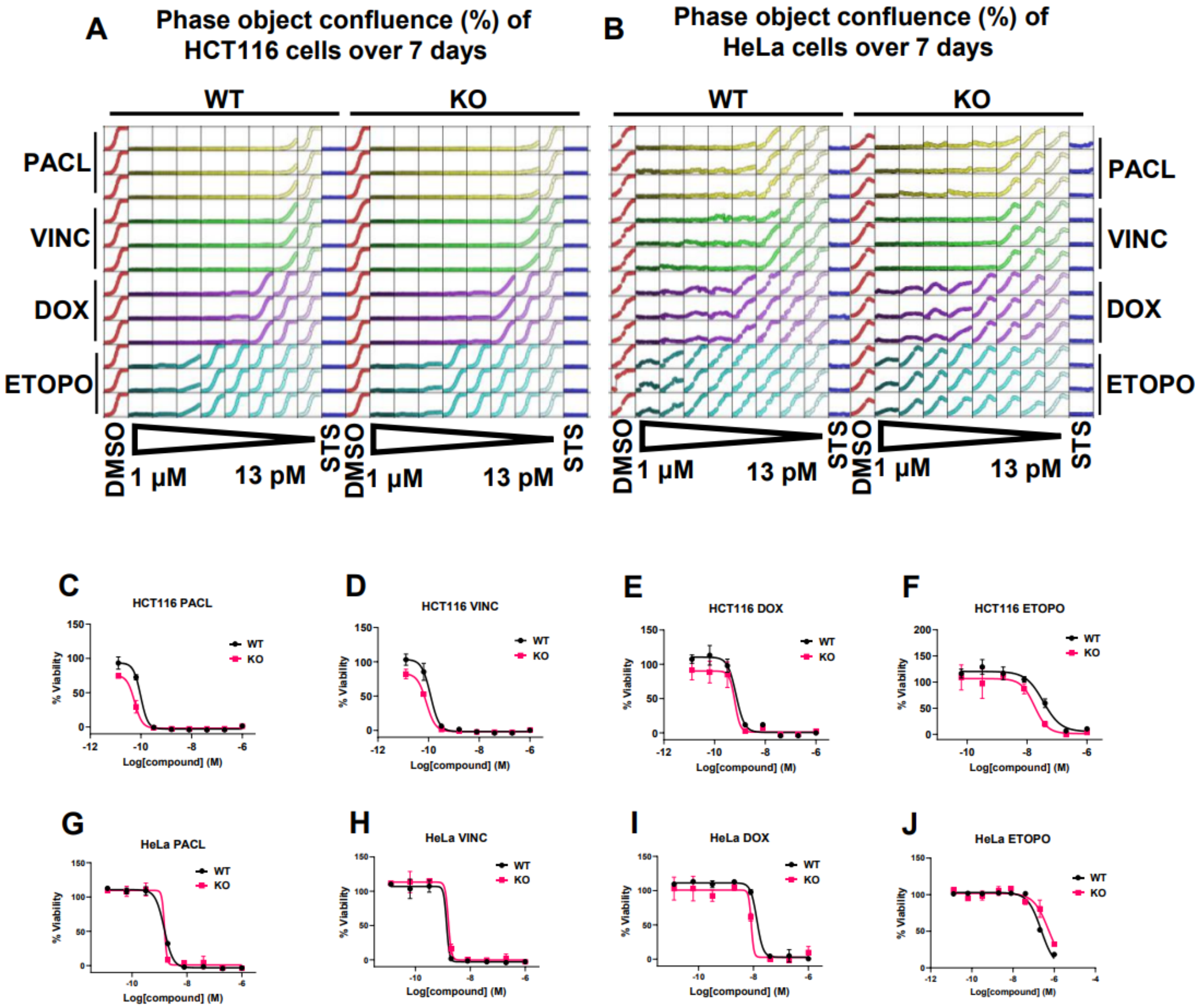

Figure S2. Live-cell analysis of phase object confluence (%) in chemotherapy-treated **A)**. WT vs. KO HCT116 and **B)**. HeLa cells on Incucyte® over 7-day period. Cell viability measured by CTG® after 7-day treatment with PACL, VINC, DOX or ETOPO in **C-F)**. HCT116 and **G-J)**. HeLa cells. Experiments **A-J** were performed in biological duplicate, with each one in technical triplicate. All data points on viability curves represent average % viability ± SD.

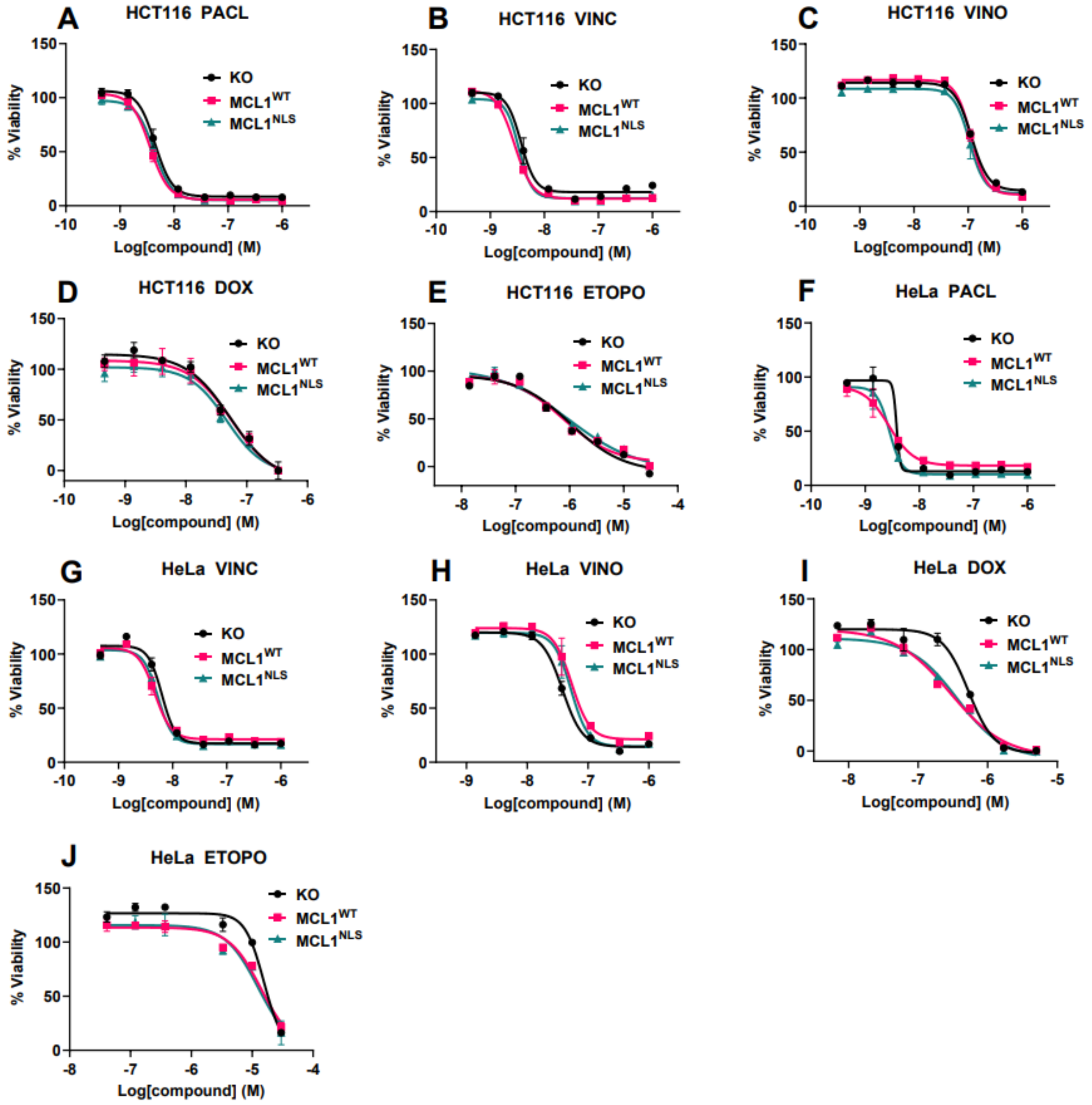

Figure S3. Cell viability assays on parental MCL1^{KO} vs. KO cell lines reconstituted with MCL1^{WT} or MCL1^{NLS}. Cell viability was measured by CTG® after 48 h treatment with PACL, VINC, VINO, DOX or ETOPO in **A-E)**. HCT116 and **F-J)**. HeLa cells. Cell viability assays were performed in biological triplicate, with each one in technical duplicate, so data points represent average % viability ± SD.

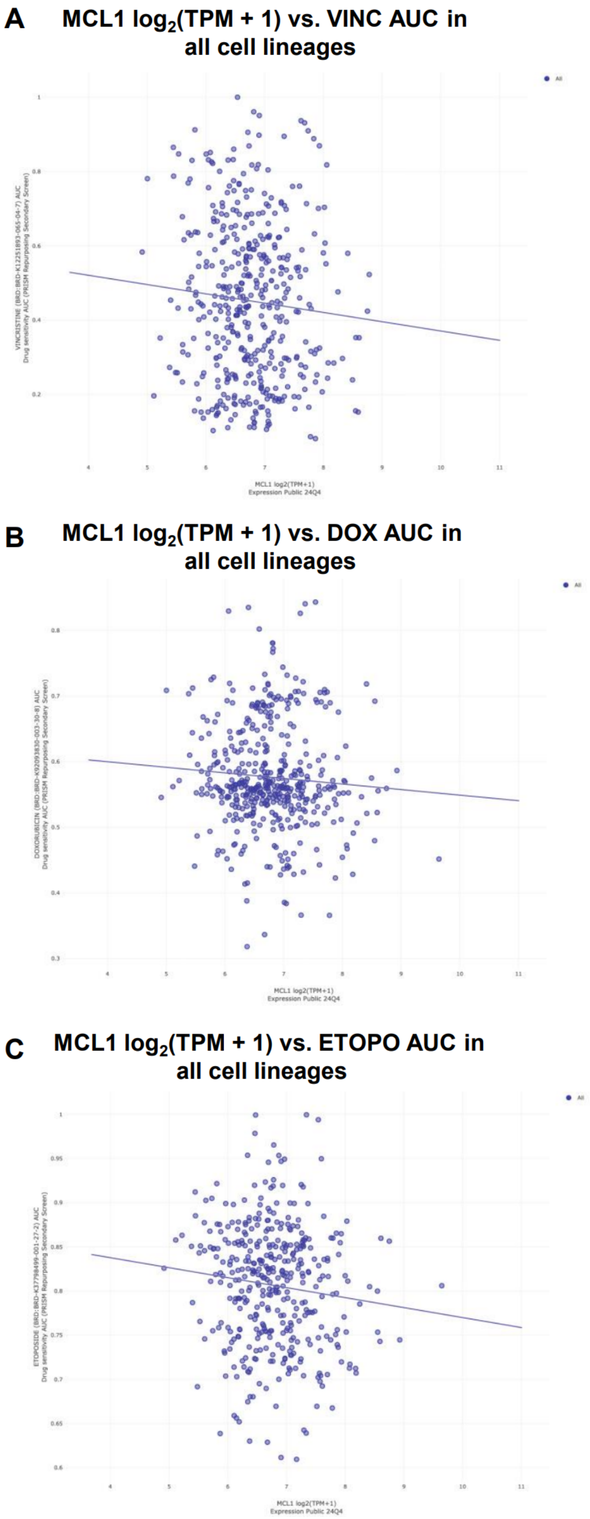

Figure S4. MCL1 RNA expression (log2(TPM + 1)) vs. AUC from viability experiments utilizing chemotherapeutic treatments. Graphs were generated using the publicly available datasets in the Broad Institute's DepMap and show the correlation between MCL1 expression and AUC in ~900 cancer cell lines in response to **A-B)**. VINC, **C-D)**. DOX or **E-F)**. ETOPO treatments. An AUC value of 1.0 represents 100% cell viability, whereas a value of 0.0 represents 0% cell viability.

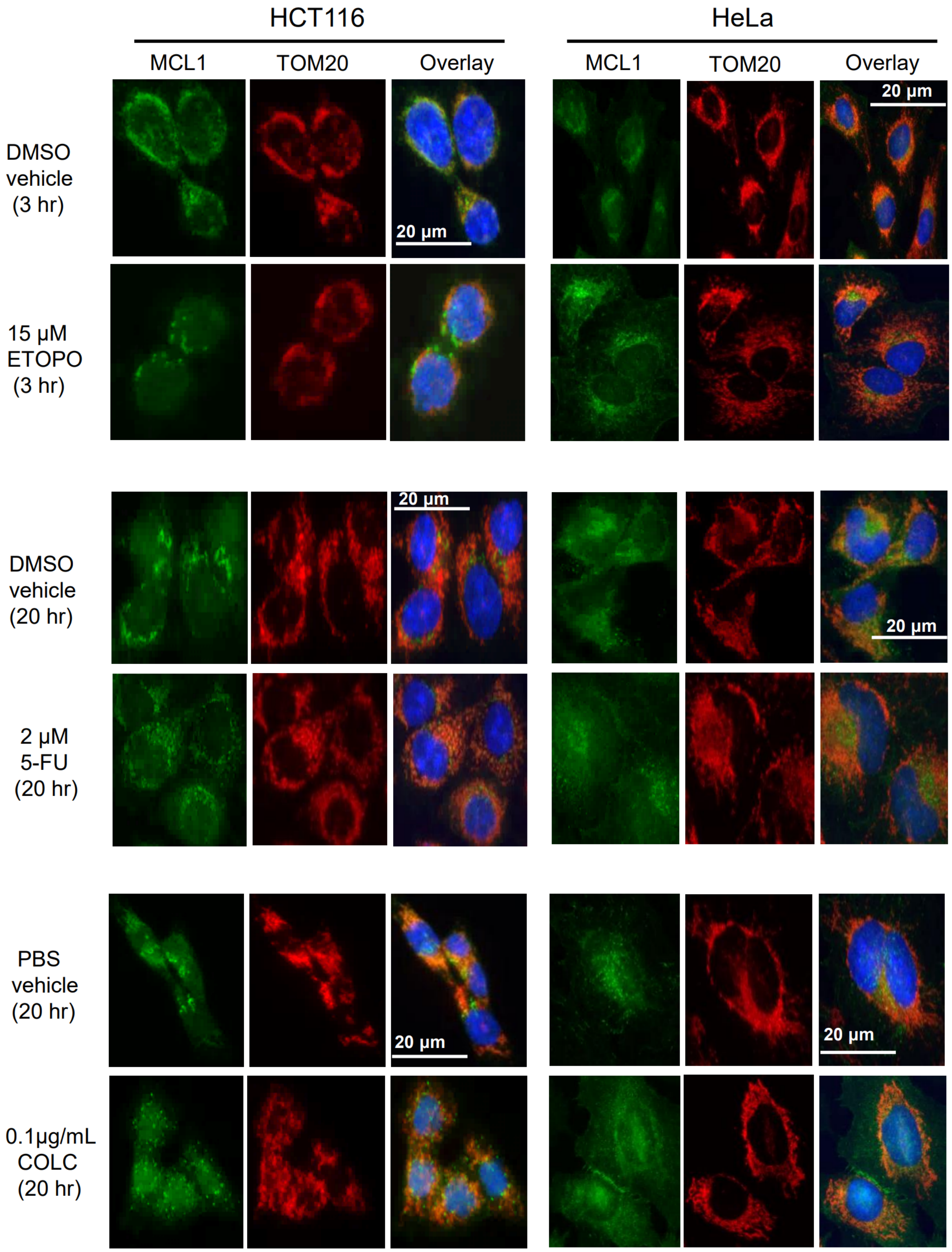

Figure S5. IF evaluation of MCL1 nuclear translocation in response to 3 hr 15 µM etoposide, 20 hr 2 µM 5-fluorouracil (5-FU) or 0.1 µg/mL colcemid (COLC) treatments in HCT116 and HeLa cells. IF images were taken at 40x magnification. Data are representative of two independent experiments.

A **MCL1^{TurboID} vs. GFP^{TurboID} HCT116 Cells**

SP > 0.8

MCL1 (SP = 1.0, FC = 488 excluded from figure)

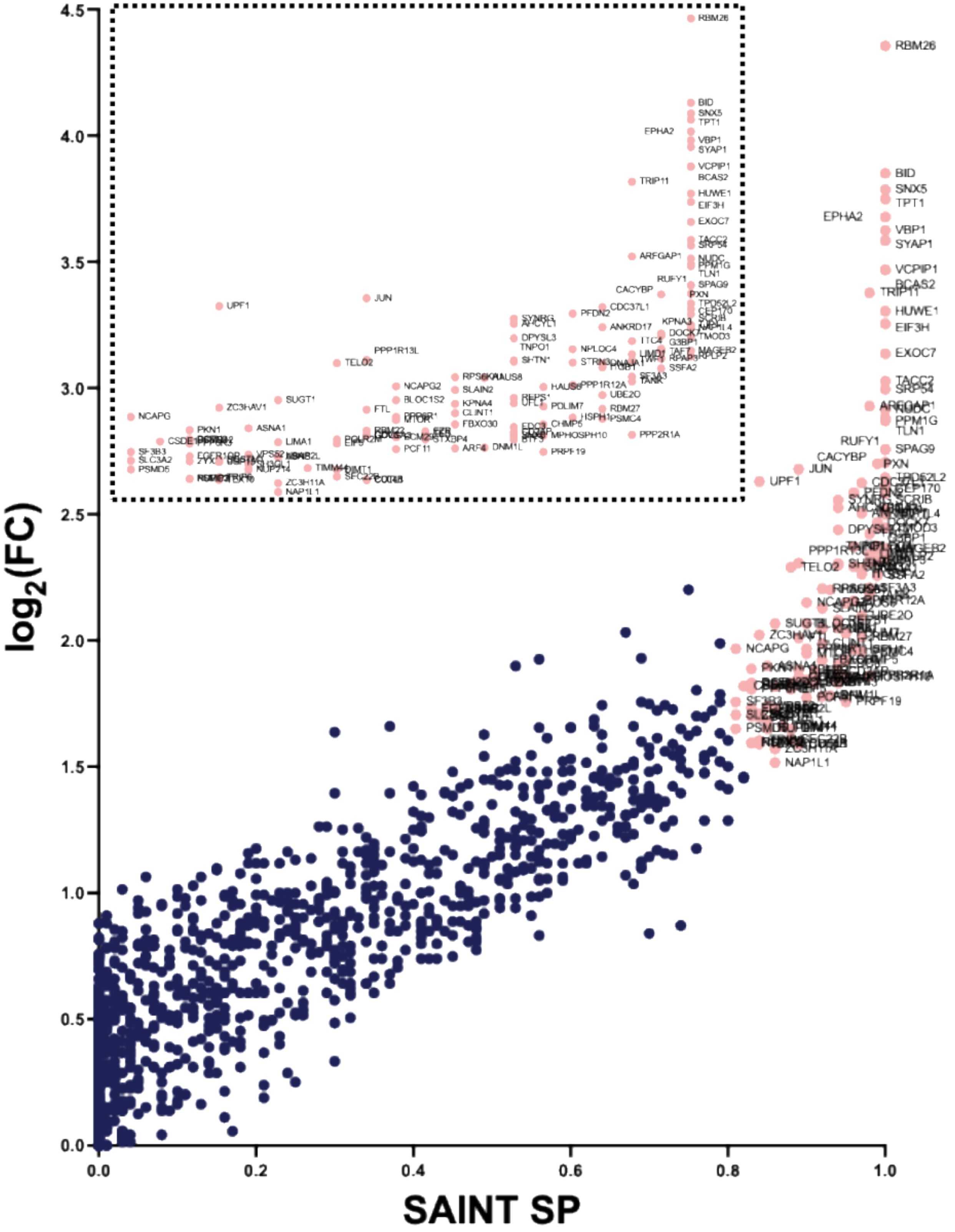

B

MCL1^{TurboID} vs. GFP^{TurboID} HeLa Cells

SP > 0.8

MCL1 (SP = 1.0, FC = 335 excluded from figure)

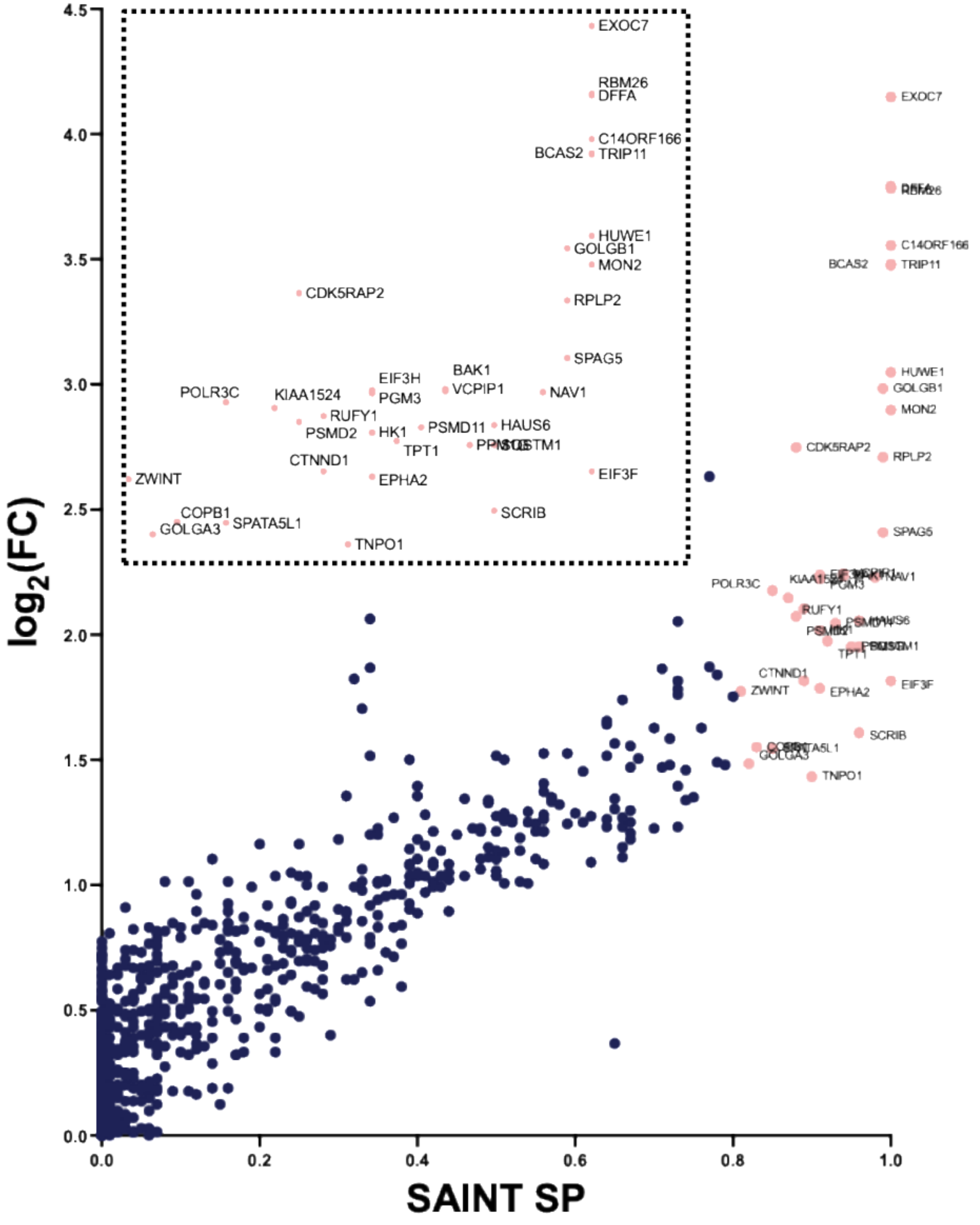

Figure S6. Volcano plots of MCL1 interactors enriched in MCL1$^{WT\text{-}TurboID}$ vs. GFP^{TurboID} with SAINT SP > 0.8 in **A)**. HCT116 and **B)**. HeLa cells. Data set shows average SP scores from three replicates.
